# Genetic Diversity and Population Dynamics of *Clinostomum* spp. Using Comprehensive Bioinformatics Approaches

**DOI:** 10.1155/vmi/6924523

**Published:** 2024-11-23

**Authors:** Sk Injamamul Islam, Mohamed H. Hamad, Wanarit Jitsamai, Channarong Rodkhum, Piyanan Taweethavonsawat

**Affiliations:** ^1^Department of Pathology, Faculty of Veterinary Science, Chulalongkorn University, Bangkok 10330, Thailand; ^2^Infectious Diseases, Department of Animal Medicine, Faculty of Veterinary Medicine, Zagazig University, Zagazig 44511, Egypt; ^3^Department of Parasitology and Entomology, Faculty of Public Health, Mahidol University, Bangkok, Thailand; ^4^Department of Veterinary Microbiology, Faculty of Veterinary Science, Chulalongkorn University, Bangkok 10330, Thailand; ^5^Parasitology Unit, Department of Pathology and Biomarkers in Animal Parasitology Research Unit, Faculty of Veterinary Science, Chulalongkorn University, Bangkok 10330, Thailand

**Keywords:** digenean parasite, haplotype diversity, metacercaria, population structure

## Abstract

*Clinostomum* species, a parasitic pathogen of freshwater fish, is widely distributed and infects various host species. Recently, the pathological effect due to *Clinostomum* metacercarial infection was described in aquaculture in Thailand; however, the global genetic diversity and population structure of this species have not been studied yet. Therefore, this study aimed to provide a detailed description of genetic diversity and population dynamics of the digenean *Clinostomum* isolated from *Trichopodus pectoralis* with globally recorded *Clinostomum* species. The species was characterized molecularly by analyzing 18S rDNA and inter-transcribed spacer biomarker genes (ITS1 and ITS2). A BLAST search discovered that the 18S rDNA and ITS sequence had a 100% sequence similarity with *Clinostomum piscidium* isolated from India and Thailand. A comprehensive analysis revealed the presence of 12 distinct haplotypes among the *Clinostomum* populations. This study suggests that distinct patterns of genetic variation were identified by analyzing molecular variance, pairwise Fst, and employing structure analysis. It was observed that a gradient of genetic variation exists within continents, characterized by higher levels within different groups and lower levels of genetic differentiation. Additionally, a notable presence of mixed haplotypes was observed. The results of neutrality testing suggest that there has been a significant expansion in the populations of *Clinostomum* in India, America, and Kenya. The discoveries from this study will provide a valuable contribution to comprehending the genetics and evolution of *Clinostomum* species. Furthermore, key findings will be essential in developing efficient management approaches to prevent and control this parasite.

## 1. Introduction

The snakeskin gourami (*Trichopodus pectoralis*, Regan 1910) is a well-liked fish for human consumption, commonly seen in Southeast Asian countries, and is recognized as a nutritious source of animal protein, beneficial for a balanced diet [1]. Aquaculture is an excellent application of this species, one of the most common air-breathing freshwater fishes of the Indo-China Peninsula [2, 3]. In Thailand, this fish species is very popular due to its high economic value, and it has emerged as one of the top five freshwater aquaculture species [4, 5]. However, one of the most significant barriers to *T. pectoralis* cultivation is the increased occurrence of parasitic infections [6]. Therefore, it is crucial to identify the parasite to comprehend its role in causing diseases.

It has previously been reported that infection by *Clinostomum* spp. has been the most serious issue with *T. pectoralis* in pond culture fields [7–10]. *Clinostomum* parasites can be detected within the abdominal cavities of infected fish, whether in a free state or attached to adipose tissue and the surfaces of visceral organs and also in the skull [11] and brain of the fish [12]. Fish-eating birds, reptiles, and (infrequently) certain mammals may have adult parasites living in their oral cavities, pharynxes, or esophagus [13, 14]. The parasites go through two different stages of development, namely, cercariae and metacercariae, within their intermediate hosts, which are both snails and fish [15]. Freshwater fish are the second intermediate host, and the infectious metacercarial stage encysts in the inner lining of the abdominal cavity and inside the muscle with the potential to cause damage to muscle tissue and internal organs [16]. The definitive hosts of *Clinostomum* parasites are typically fish-eating birds (e.g., herons, egrets, and cormorants) where the parasites mature, reproduce, and release eggs through the host's feces [17]. After ingestion, *Clinostomum* metacercaria first penetrates the intestinal tissue of the bird's host and then emerges and migrates to the anterior part of the digestive system to mature into an adult. In cases of heavy infection, the emergence of immature parasites from the intestinal tissue can lead to mortality [18]. In fish, the severity of the infection determines the range of consequences, which can vary from decreased fish growth and reproductive efficiency to the appearance of hemorrhagic signs on the body surface and even mass death [19, 20]. In addition, some *Clinostomum* parasites affect humans as a zoonotic pathogen and can cause human laryngitis [21].

To ensure effective therapy and control, it is essential to accurately diagnose and identify the parasite at the species level using molecular techniques. The genus *Clinostomum* has undergone multiple revisions in its taxonomy due to the presence of similar morphological characteristics and a lack of clear boundaries among its species [22, 23]. For the effective taxonomic identification of parasites, the ribosomal DNA (rDNA) genes of the larger subunit (LSU or 28S), smaller subunit (SSU or 18S), and the inter-transcribed spacer (ITS1 and ITS2) regions have been utilized as molecular markers [24, 25].

The comprehension of genetic differentiation and population structure in protozoan parasites is crucial for assessing their evolutionary processes, spatiotemporal dynamics, and genetic exchange and formulating efficient measures to mitigate disease transmission [26]. Molecular markers, including 18S rRNA, mitochondrial DNA, microsatellite loci, and single nucleotide polymorphisms, are frequently utilized in scientific investigations to examine genetic diversity. These markers offer valuable insights into reproductive modes and molecular sequences derived from samples, enabling the elucidation of the evolutionary history of populations [27]. Several genetic diversity studies of the metacercaria of *Microphallus piriformes* [28], *Posthodiplostomum minimum* [29], and *Echinostoma mekongki* [30] have currently been reported. Earlier studies have provided evidence indicating the presence of significant genetic variability among populations of trematode metacercaria. Previous studies have offered insights into the morphology, phylogenetic, and demographic divergence of *Clinostomum* spp., which are capable of parasitizing fish, birds, and humans [10, 17, 31, 32]. Molecular detection of *Clinostomum* spp. has been documented globally [17, 33, 34], but comprehensive investigations into the genetic diversity of *Clinostomum* spp., utilizing both morphological and molecular analyses, are still outstanding.

In contrast to our information on the morphology of clinostomid parasites in Thailand, our understanding of their genetic diversity and population dynamics is lacking. It is now common and effective to employ sequence data to confirm the taxonomic identities of parasites and to identify immature and larval forms of parasites and explain their life cycles [35, 36]. Therefore, to explicitly recognize them, the current research focused on genetically identifying metacercariae belonging to the family Clinostomidae from the freshwater aquaculture species *T. pectoralis* in Thailand. Furthermore, this study sought to clarify the genetic variation, population changes, and population organization of *Clinostomum* populations across multiple continents, namely, Asia, Australia, Americas, Africa, and Europe, by utilizing the 18S rRNA gene.

## 2. Materials and Methods

### 2.1. Ethics Statement

The research protocols for this study have been approved by Chulalongkorn University in Thailand, ensuring compliance with the institution's animal ethical requirements (protocol number: IBC 2231038).

### 2.2. Collection of *T. pectoralis* and Parasite Specimens

Between January and July 2023, 262 live specimens of *T. pectoralis*, aged between 4 and 8 months, were gathered from 12 aquaculture farms located in Samut Prakan, Samut Songkhram, Samut Sakhon, and Kanchanaburi provinces of Thailand as these provinces are the primary providers of the market, with a significant number of *T. pectoralis* farms. The fish lengths varied from 13.28 to 20.00 cm, while their weights ranged from 58 to 252 gm. The fish were brought to the Parasitology Unit of the Faculty of Veterinary Science, Chulalongkorn University, Thailand, and maintained in aquaria under proper aeration. The fish were euthanized with tricaine methanesulfonate (250 mg/L) [37]. A standard protocol was followed to examine the fish for parasites [38]. Initially, a necropsy was conducted, and the visible body cavity was visually inspected for parasites. Following this, the liver, kidney, spleen, and intestine were carefully examined for parasitic presence. Parasites were observed attached to the surface of the intestine, spleen, and liver, but no parasites were detected within the internal tissues of these organs. Once the parasites were counted, specific samples underwent further analysis for molecular examination.

### 2.3. DNA Extraction and PCR Amplification

All metacercarial specimens were rinsed in normal saline before being preserved in 70% ethanol until DNA extraction. Genomic DNA from samples was extracted using a NucleoSpin DNA extraction kit, following the manufacturer's instructions (NucleoSpin, Germany). The primer pairs listed in Table 1 were used to amplify the sequences of 18S rDNA and ITS1-5.8S-ITS2. PCR was performed by following our previous study [10]. The final PCR products were then separated using a 1.5% agarose gel and the expected product from PCR was purified using a commercial kit manual (NucleoSpin Gel and PCR Clean-up, Macherey-Nagel, Düren, Germany) and sequenced by a commercial service (Celemics, Korea).

### 2.4. Sequence Analysis and Construction of Phylogenetic Tree

The 18S rRNA and ITS1-5.8S-ITS2 sequences were subjected to analysis using the NCBI BLASTN program (https://www.ncbi.nlm.nih.gov/BLAST/). The resulting sequences were subsequently deposited in GenBank with the accession numbers OP793987, OP793988, OP793989, OP793990, OP793991, OP793992, OP793993, and OP793994 from 18s rDNA and OP782661 from ITS1-5.8S-ITS2 gene. Phylogenetic analysis was performed based on the 18s rDNA and ITS1-5.8S-ITS2 gene dataset after performing BLASTN and retrieving the sequence from the GeneBank. The details of the sequences are listed in Tables 1 and 2 in Supporting file. Multiple sequences were aligned using the ClustalW multiple alignments feature of MEGA 11 with the elimination of the sequences missing annotation, such as information on the host or location, and shortened sequences [34]. For each dataset (18S rDNA, ITS1, and ITS2), phylogenetic trees were constructed using MEGA 11 [39] and visualized by ITOL (https://itol.embl.de/), an interactive web tool for phylogenetic tree visualization. Before constructing the phylogenetic tree, the best model fit for the analysis was determined by using jMODELTEST 2.1.7 [40] which is a tool to carry out a statistical selection of best-fit models of nucleotide substitution, followed by examining using maximum likelihood (ML) methods [41, 42]. Potential species were separated using clustering in the ML approach of the optimal nucleotide substitution model, as predicted by the “Kimura 2 parameter model.” It was determined that a gamma distribution of rates and proportion of invariant sites (GTR + G + I) would provide the best fit for the ML analysis data [43]. Using the bootstrap approach with 1000 replicates, it was determined that the internal branches in all trees were valid [43].

### 2.5. Genetic Diversity, Haplotype Data, and Statistical Analysis

Genetic factors, such as the haplotype number, haplotype diversity (Hd), and nucleotide diversity, were assessed using DnaSP 5.1 [44]. The dataset for this analysis of 18s rDNA of all the newly obtained sequences and other sequences from the Gene Bank is listed in Supporting Table 3. The genetic differentiation between pairs of populations was assessed by conducting pairwise Fst and Nst calculations using the DnaSP 5.1 software. The Fst values serve as an indicator of the degree of genetic differentiation. A low level of genetic differentiation is seen when the Fst value falls within the range of zero to 0.05. Moderate genetic differentiation is indicated by Fst values ranging from 0.05 to 0.15, while strong genetic differentiation is observed when Fst values range from 0.15 to 0.25 [45, 46]. The positive values of Tajima's D [43] and Fu's Fs [44] show the absence of departure from neutrality, which is characteristic of populations that are not undergoing any significant changes. On the other hand, negative values of both statistics suggest that the populations have had recent expansion or have been subjected to purifying selection. The aforementioned studies excluded populations in China and Israel nations that consisted of just one sample. In order to assess the associations between haplotypes, a median-joining network was constructed using the PopArt 1.7 software [47].

The present study used the hierarchical analysis of molecular variance (AMOVA) to evaluate the genetic variation between and within populations of *Clinostomum* and pairwise mismatch distribution analysis was conducted to assess neutrality using Arlequin 3.5 software [48]. Structure v2.3.4 was used to conduct population genetic structure [49] using the admixture ancestry model. Each value of *K* (*K* = 2 − 10) was run with 3 iterations based on the Bayesian Markov Chain Monte Carlo approach. The appropriate number of clusters (K) was determined using the online tool Structure Harvester, using 1K iterations [50, 51]. To analyze the beta diversity distances between each sequence a principal coordinate analysis (PCoA) was conducted by PAST v4.03 [52] due to the number of missing data and characters among sequences. The Mantel test was used to assess the relation between genetic distance and geographical distance, pairwise permutational multivariate analysis of variance (PERMANOVA) was conducted between the continents to assess the association of the population [53], and pairwise genetic differentiation and Fst value were visualized by using R packages [54].

## 3. Results

### 3.1. Sequence and Phylogenetic Analysis

PCR amplification was used to generate a partial segment of the 18S rDNA gene and a 339-bp contig was formed after sequencing the PCR product. Using *Schistosoma spindale* as an outgroup, a phylogenetic tree based on 18S rDNA was constructed. This tree included various *Clinostomid* trematode isolates obtained from different geographic locations. This study also used the Kimura 2 parameter model and ML method, as shown in Supporting file Tables 4 and 5, to estimate the genetic differences necessary for evolutionary biology research. To determine divergence and distances, the Neighbor-Join and BioNJ algorithms were performed on a matrix of pairwise distances calculated using the maximum composite likelihood (MCL) approach, and the topology with the highest log-likelihood value was selected as the initial tree for the heuristic search.

**Figure 1:**
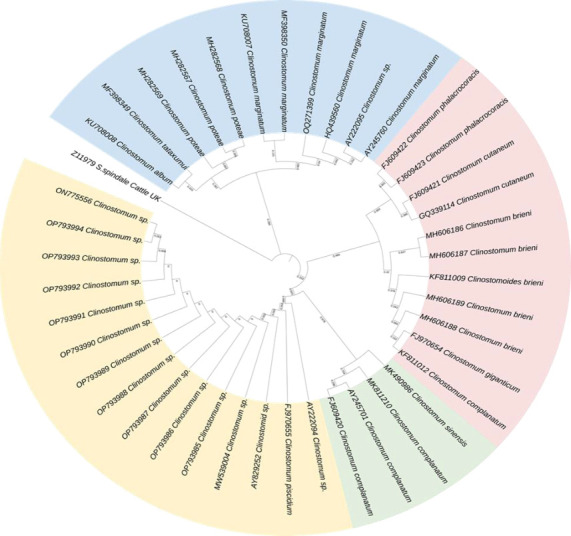
Evolutionary relationships between *Clinostomid* determined based on partial 18S rDNA sequences using the maximum likelihood method. The percentages of replicate trees in which the related taxa are grouped in the bootstrap test (1000 replicates) are displayed above the branches.

On the other hand, the phylogenetic tree indicated that our isolate had 99% similarity with *Clinostomum piscidium* (India), *Clinostomum* sp. (Australia), *Clinostomum* sp. (Thailand), and *Clinostomid* sp. (United States), each representing distinct isolates from these regions (Figure 1) (Supporting file Table 1). Another group containing *C. complanatum* from China, *C. complanatum* from the USA, and *C. complanatum* from Israel under the same clade showed 97% similarity based on the BLAST results. According to the sequence identity matrix of clinostomids (Supporting file Table 1), our isolate was 100% identical to the Australian, Thailand, and USA isolates of *Clinostomum* sp. and 98.93% identical to the Indian isolate of *C. piscidium*, which further supports the phylogenetic tree.

**Figure 2:**
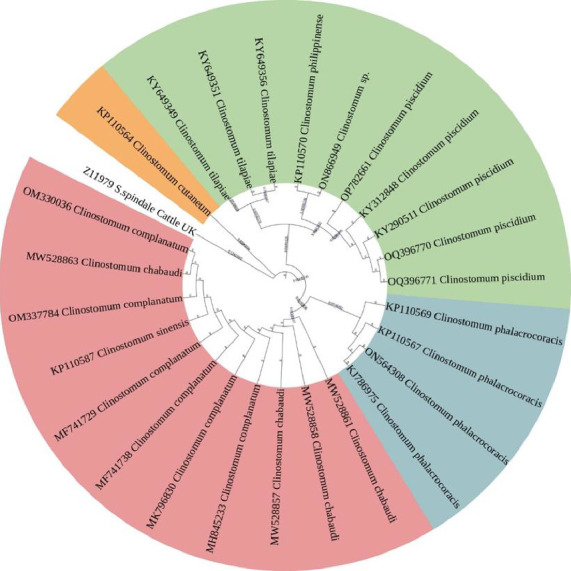
ITS phylogenetic tree constructed by maximum likelihood displays *Clinostomum piscidium* (OP782661) from *T. pectoralis* clustering with other *C. piscidium*. GenBank accession numbers are provided before species names, with the numbers on the nodes reflecting bootstrap confidence levels.

Furthermore, a 801-bp fragment of the *C. piscidium* ITS1-5.8S-ITS2 genes was uploaded to GenBank to facilitate molecular research (accession number: OP782661). The present partial sequence showed 100% homology with *C. piscidium* isolated from India and Thailand. In addition, for the phylogenetic analysis, sequences with lower similarity (96%–99%) based on the BLAST results were also employed (Supporting file Table 2). The isolated *C. piscidium* from *Colisa fasciata* and the current species were closely linked based on phylogenetic analysis and were placed in the same clade with high bootstrap values (Figure 2).

### 3.2. Genetic Diversity and Haplotype Network

Table 2 shows the identification of 12 distinct haplotypes from a collection of 41 18s rDNA sequences obtained from the *Clinostomum*. The alignment of these sequences, which consisted of 339 base pairs, revealed 40 variable sites. The most common and geographically widespread haplotypes, Hap1 and Hap10, were found in 15 and 6 *Clinostomum* isolates, respectively. These isolates were collected from 7 different countries across 5 continents, as indicated in Table 2.

**Figure 3:**
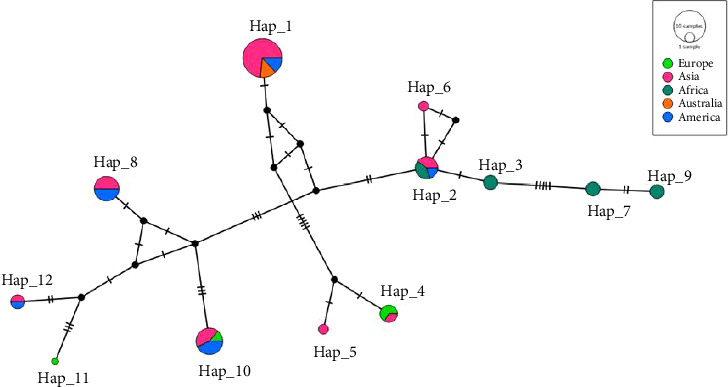
Median-joining haplotype network of *Clinostomum* populations in Asia, Australia, America, Africa, and Europe. Haplotype Hap1 contains 15 *Clinostomum* isolates from Thailand, India, Australia, and the USA. The overall size of the circular shape is indicative of the relative occurrence rate of each haplotype. Each of the colors of the dots symbolizes distinct haplotypes originating from diverse populations.

The haplotype network analysis was conducted on several populations of *Clinostomum* spp. originating from Asian countries is shown in Table 3 and Figure 3. The Hd ranged from 0.0 to 1, while the nucleotide diversity varied from 0.0 to 0.37635. The United States exhibited the highest Hd, while India showed the greatest nucleotide diversity. On the other hand, Thailand, Australia, the Republic of Congo, and South Africa had the lowest levels of both haplotype and nucleotide diversity, as they consisted of only one haplotype (Hap1). The analysis revealed a lack of significant sequence divergence and the presence of distinct mutations among the populations.

### 3.3. Neutrality and Demographic Analysis

Both the United States and India displayed statistically significant negative values for Tajima's D and Fu's Fs, suggesting a recent expansion in population size. When considering all the Asian populations collectively, Tajima's D and Fu's Fs values were strongly negative, indicating population growth. This was further supported by the analysis of pairwise mismatch distribution, as presented in Supporting file Table 6. However, the populations of Thailand, Australia, and the Republic of Congo did not show any signs of growth, as indicated by the results of Tajima's D and Fu's Fs analyses in Table 3.

### 3.4. Population Genetic Differentiation

**Figure 4:**
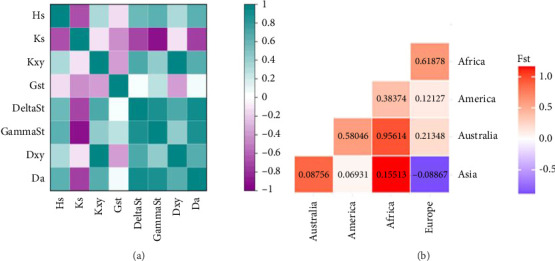
(a) Correlation of pairwise genetic differentiation parameters among the continents and (b) pairwise Fst value of within and between the continents.

**Figure 5:**
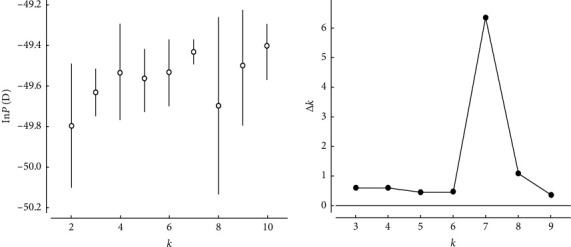
Changes of the ln*P* (D) (mean ± SD) for *K*-values ranging from 2 to 10 and the relationship between delta *K* and *K* values. The maximum delta *K* value at *K* = 7 indicating the true number of clusters is seven.

The global population differentiation is described in Table 4. The findings of this study revealed that there was variability in genetic differentiation across all population pairs. The Fst values ranged from −0.08867 to 0.95614, while the Nst values ranged from −0.01198 to 0.9519, as shown in Table 4.  Figure 4(a) and Supporting file Table 7 explore the genetic differentiation and diversity among *Clinostomum* parasites. Within each continent, there is notable genetic diversity (Hs), indicating diverse genetic backgrounds within the *Clinostomum* populations. The differences in synonymous (Ks) and nonsynonymous (Kxy) substitutions suggest the influence of natural selection on genetic variation. The genetic differentiation among populations within continents (Gst) is moderate, while differentiation between subpopulations within populations (DeltaSt) and populations within continents (GammaSt) is relatively low. Genetic distance (Dxy) between *Clinostomum* populations from different continents reflects historical migrations and interactions. In comparison to other groups, the African population exhibited the greatest genetic divergence, as shown by the Fst and Nst values presented in Table 3 and Figure 5.

The Fst and Nst values were also calculated for comparisons between samples from Asia and those from America, Africa, Australia, and Europe. The analysis of the data indicated that Africa exhibited the greatest degree of difference, as shown by a Fst value of 0.15513 and a Nst value of 0.54927. Conversely, Europe had the lowest level of differentiation, with a Fst value of −0.08867 and a Nst value of −0.01198, as shown in Table 4.

The AMOVA revealed that the majority of genetic variation was seen across populations, as shown in Table 5. The hierarchical AMOVA revealed that most of the genetic variation was seen across populations. This finding is further confirmed by the low Fst values, which were not statistically significant (Table 5). The results of this study demonstrated a lack of significant genetic difference and a substantial level of gene flow across groups.

The findings from the PERMANOVA study indicate significant dissimilarity across all pairs of continents (Table 6). Asia and Australia have a comparatively lower dissimilarity coefficient of 0.4103, indicating a higher degree of similarity in terms of genetic or ecological characteristics. On the other hand, it is worth noting that there exists a notable dissimilarity (3.202) between Asia and America, suggesting a substantial difference between these two continents.

### 3.5. Population Structure Analysis

**Figure 6:**
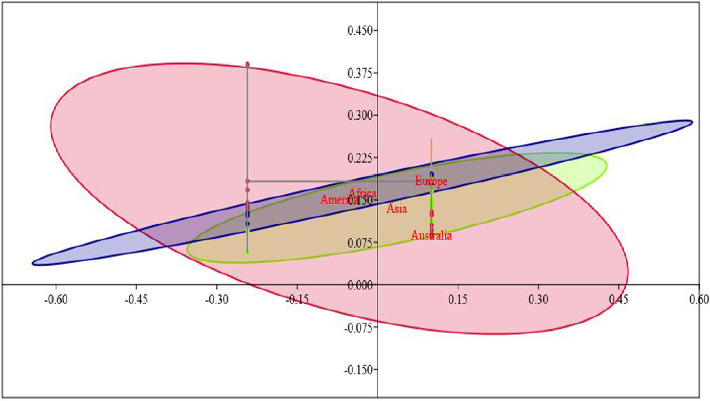
Principal coordinate analysis of the population genotypic data.

The examination of population structure uncovered distinct mixed haplotypes, indicating a limited degree of differentiation among Asian populations. Similar findings were noted in the genetic composition of *Clinostomum* populations across Asia, Australia, Africa, America, and Europe (data not presented). Bayesian clustering analysis identified seven distinct clusters (*K* = 7, see Figure 5). PCoA was utilized to explore the dataset dimensions of *Clinostomum,* effectively maintaining the dissimilarities among data points (Figure 6). The Mantel test showed no significant relationship between genetic and geographic distances among global individuals (*p*=0.49451, see Supporting file Table 8).

## 4. Discussion

*Clinostomid* parasites, potentially zoonotic and found worldwide, have not been extensively researched using molecular taxonomic methods to describe Hd and population dynamics. This research focused on DNA sequencing data, genetic diversity, and population structure analysis for future research essential for accurately describing closely related taxa of *Clinostomum* spp.

In this study, PCR amplification with rDNA markers successfully amplified parasites isolated from fish hosts, supporting previous research on the molecular characterization of trematodes found in freshwater snails (first intermediate hosts) [55]. The analysis of 18s rDNA molecular distances in this study indicated a close relationship between the 10 sequences and *C. piscidium* isolated from fish species. Similarly, all sequences from this study showed a relatively short genetic distance when compared to *C. complanatum* and *Clinostomum* sp. from India and Australia, respectively. Moreover, the comparison of genetic distances using the ITS biomarker gene further confirmed the close relationship between the sequences from this study and *C. piscidium* from India [17] and Thailand [10]. Previously, one study tried to validate *Clinostomum marginatum*, utilizing the internal transcribed spacer 1 (ITS1) region, and compared it to adult and larval specimens from different hosts and geographical regions [23]. Based on 18s rDNA sequence, our isolated *Clinostomum* sp. from the host *T. pectoralis* showed genetic resemblance to *C. piscidium* from two different fish hosts isolated from India and Thailand. In addition, according to the phylogenetic tree, the *Clinostomum* sp. (AY222094) isolated from Murray cod (*Hypseleotris galii*) from Australia demonstrated a very strong association with the 99% sequence similarity matrix in BLASTN. Previous findings on *C. complanatum* from China, the United States of America, and Israel were all placed in a different group within the same clade as our isolate, which shared 97% of their sequence similarities. In conclusion, the 18S rDNA gene–based phylogenetic tree and sequence identity matrix (Supporting Table 1) were able to identify *C. breini*, *C. marginatum*, and *C. tataxumui* and place them in a different clade, which corroborated the conclusions of earlier research [56–58]. The ITS gene phylogenetic tree presented in Figure 3 highlights that our *C. piscidium* isolate from *T. pectoralis* grouped into the same cluster as an Indian isolate of *C. piscidium*.

In our work, we report the first genetic diversity and population structure data of *Clinostomum* metacercariae comparing isolated parasites from Thailand and available sequences of other *Clinostomum* spp. from Gene Bank. Thus, the molecular analysis along with genetic diversity and results from population structure allowed us to distinguish *Clinostomum* parasite from different host sources and geographical locations. According to this study, the nucleotide diversity in India was found to be 0.37635, while in Thailand, it was 0.0. These findings indicate a significant difference in nucleotide variation among *Clinostomum* populations in Asia, with higher values compared to *Opisthorchis felineus* [59], but comparatively similar to other metacercariae of trematode genus *Crepidostomum*, *Tylodelphys*, *Plagiorchis*, *Echinoparyphium*, *Trichobilharzia*, *Radix* spp., and *Pisidium* spp. [60]. Nevertheless, a low level of nucleotide diversity was found in *Clinostomum* spp. in America with a higher Hd [61]. The haplotype analysis results revealed a significant level of genetic variety, particularly in Asian and American nations, except in some regions. Thailand exhibited an apparent shortfall in Hd, indicating a potential correlation between the restricted genetic variety among its populations and limited gene flow. However, the high diversity of trematode metacercaria is found in freshwater snails in Bangkok, Thailand [30]. Compared to other *Clinostomum* species like *Clinostomum marginatum*, India had the highest Hd while America had a slightly lower Hd of 0.80556 [61]. The size of a population is a crucial determinant in influencing the genetic variety of a parasite [62]. Consequently, this research observed a correlation between lower Hd and smaller effective population size.

The impact of the demographic bottleneck on the genetic diversity of *Clinostomum* populations is a significant aspect to consider. The results of neutrality tests indicate that the populations from Asia and four other continents have deviated from neutrality. This suggests that these three populations have likely seen recent expansion, maybe due to factors such as bottlenecks or the founder effect [63]. Moreover, the observed populations in India, America, and Kenya may experience an overabundance of rare haplotypes due to their rapid increase in population.

Gene flow serves a vital role in the evolutionary dynamics of populations, exerting a dual effect by augmenting genetic diversity within populations while concurrently diminishing genetic differentiation across populations [64, 65]. This research observed that a significant proportion of genetic diversity among populations in India, America, and Kenya was identified, suggesting a substantial level of gene flow among these groups, similar to that has been seen in *E. mekongki* [30]. However, in various geographic regions, there was a notable disparity in the level of gene flow seen among metacercariae of *M. piriformes* [28]. The presence of high gene flow was further confirmed by the observation of low pairwise Fst values, indicating a limited degree of genetic difference among the groups. The findings of this study provide evidence that genetic interchanges took place across these populations and maybe associated with the rapid geographical expansion of migratory hosts, namely, birds [66]. Nevertheless, a notable disparity in genetic variation was identified between the population of the United States and populations in India, perhaps indicating a correlation with restricted genetic interchanges within the Asian region. The United States of America is recognized as a country with a substantial land area, characterized by a temperature and environment conducive to the survival of *C. album* n. sp. and *C. marginatum*. These particular species serve as appropriate hosts for the great egret *Ardea alba* [67], so it may be deduced that the distinct biological system of America hindered the dissemination of definitive hosts and genetic recombination of *Clinostomum* spp. with other populations, hence explaining the greater genetic differentiation seen in the American population. At a broad level, the observation of the greatest Fst value between the American and Indian populations suggests a limited exchange of genetic material between these groups spanning across continents. The genetic divergence between the American and Indian populations was found to be minimal, perhaps indicating a connection to the Asian genotype of *Clinostomum* spp. that is native to America [68]. The findings of this study align with a previous investigation on trematode species, wherein no or minimal genetic differentiation was observed among populations from adjacent countries within the same continent. However, notable genetic differentiation was detected across different continents, which can be attributed to the geographical isolation resulting from distance-based patterns [30].

In the absence of a significant association between genetic and geographical distances across *Clinostomum* populations (Mantel test, *P*=0.49451), our analysis revealed the presence of seven distinct subgroups distributed worldwide. These findings were validated by Bayesian analysis (*K* = 7) and PCoA of beta diversity, considering the limitations imposed by missing data and sequence variability. The evident admixture of haplotypes suggests that the differentiation among these populations was not fully accomplished, and the same observations have been seen in *Theileria annulata* in Asia [69]. Regardless of the presence of ecological and geographical obstacles, the exchange of genetic material between these populations resulted in an intricate population configuration, likely influenced by the regular migration of avian carriers of mature *Clinostomid*, as well as the movement of snails and other secondary hosts such as fish or mollusks [56]. Hence, it is essential to conduct more research to ascertain the impact of migratory bird activities on the dissemination of *Clinostomum* spp. Several significant elements contribute to the development of intricate and distinctive genetic structures and epidemic patterns within *Clinostomum* populations. These aspects include transmission intensity, parasite–host coevolution, geographic and ecological segregation, and selection pressure.

## 5. Conclusion

*Clinostomum* spp. are parasites that have a significant economic impact on freshwater fishes, snails, and birds worldwide. Accurate identification of these parasites at the genus and species levels is crucial for controlling and preventing their spread. Previous studies have not investigated the molecular identification and genetic diversity of *Clinostomum* spp. in *T. pectoralis* in Thailand. This study used 18s rDNA and an ITS biomarker gene to determine the species of the parasite. Phylogenetic analysis of the gene sequences showed that our sequence was similar to *C. piscidium* found in India. The study also examined the genetic diversity of *Clinostomum* spp. and it was found that there is little genetic differentiation between parasites on different continents, with significant gene flow and variety within populations spanning different continents. In addition, neutrality tests indicated that the populations studied have experienced demographic growth. This study completely focuses on 18S rDNA and ITS biomarker genes, which may not fully capture the genetic diversity of *Clinostomum* spp. The lack of mitochondrial biomarkers in available databases further limits the accuracy of genetic diversity analysis. Additionally, the influence of environmental factors on the genetic diversity and population structure of these parasites was not explored. However, the findings from our study surely contribute to our understanding of the genetic variety and population structure of *Clinostomum* spp. and can be used to develop more effective techniques for managing diseases that pose a threat to public health.

## Figures and Tables

**Table 1:** Primer pairs used in the present study.

Molecular marker	Primer	Sequence (5′-3′)	Size (bp)	Reference
18s rDNA	Forward	ATTCCGGAGGGAGCCCTG	395	[10]
	Reverse	ATCAACCCAGTCAGCACCC		

ITS1-5.8S rDNA-ITS2	Forward	CACCGCCCTGGCGTAATA	801	[10]
	Reverse	CGACACTTCGAACGATTTCTAGA		

**Table 2:** Geographic origin of *Clinostomum* spp. isolates and the number of haplotypes based on 18s rDNA.

Country name	Continent	No. of sequence	No. of haplotypes
Thailand	Asia	10	1; Hap1(10)
Australia	Oceania	2	1; Hap1(2)
USA	North America	9	4; Hap1(2), Hap8(3), Hap10(3), Hap12(1)
India	Asia	5	4; Hap1(1), Hap2(2), Hap6(1), Hap10(1)
Republic of Congo	Africa	2	1; Hap2(2)
South Africa	Africa	2	1; Hap3(2)
Kenya	Africa	4	2; Hap7(2), Hap9(2)
Mexico	North America	2	2; Hap10(1), Hap11(1)
Italy	Europe	3	2; Hap4(2), Hap10(1)
China and Israel	Asia	2	2; Hap5(1, China), Hap4(1, Israel)
Total		41	12

**Table 3:** Genetic parameters and neutrality tests of *Clinostomum* species populations.

Locations	Number of sequences	Hd	*π*	*D*	Fs
Thailand	10	0	0	0	0
Australia	2	0	0	0	0
USA	9	0.80556	0.11011	−2.8587⁣^∗∗∗^	−6.057⁣^∗∗^
India	5	1	0.37635	−1.9294⁣^∗^	−4.3824⁣^∗∗^
Republic of Congo	2	0	0	0	0
South Africa	2	0	0	0	0
Kenya	4	0.66667	0.00399	−0.5456	−1.6052
Mexico	2	1	0.14671	n.d.	n.d.
Italy	3	0.66667	0.10379	1.2252	0.2409

*Note: π*, nucleotide diversity; D, Tajima's D; Fs, Fu's Fs.

Abbreviations: Hd, haplotype diversity; n.d., not determined.

⁣^∗^*p* < 0.05.

⁣^∗∗^*p* < 0.01.

⁣^∗∗∗^*p* < 0.001.

**Table 4:** Pairwise Nst and Fst values among *Clinostomum* spp. populations of different continents.

Continent	Asia	Australia	America	Africa	Europe
Asia	—	0.08756	0.06931	0.15513	−0.08867
Australia	0.06728	—	0.58046	0.95614	0.21348
America	0.22971	0.57836	—	0.38374	0.12127
Africa	0.54927	0.9519	0.38301	—	0.61878
Europe	−0.01198	0.23529	0.12194	0.61517	—

*Note:* Nst values are above the diagonal, and Fst values are below the diagonal.

**Table 5:** Analysis of molecular variance (AMOVA) among and between each continent.

Source of variation	d.f.	Sum of squares	Variance components	Percentage of variation	*F* statistics
Among populations	4	53.527	1.52009 Va	39.64	
Between populations	36	83.327	2.31464 Vb	60.36	Fst = 0.39640
Total	40	136.854	3.83473		

**Table 6:** Pairwise PERMANOVA analysis between the continents.

Continent	Asia	Australia	America	Africa	Europe
Asia	—	0.4103	3.202	0.9565	0.6194
Australia	0.4103	—	1.714	0.96	0.6194
America	3.202	1.714	—	0.2769	2.6
Africa	0.9565	0.96	0.2769	—	1.473
Europe	0.6194	0.6194	2.6	1.473	—

## Data Availability

The data that support the findings of this study are available in the supporting information of this article.

## Supporting Information

Table 1: sequence identity matrix (%) among the clinostomids based on the partial sequence of 18S rDNA. Table 2: sequence identity matrix (%) among the clinostomids based on the partial sequence of ITS gene. Table 3: the sequences of *Clinostomum* spp. used in this study for genetic diversity analysis. Table 4: 18s rDNA genetic distances of the sequence used in the study. Table 5: ITS genetic distances of the sequence used in the study. Table 6: the demographic expansion supported by the pairwise mismatch distribution analysis. Table 7: pairwise genetic differentiation among *Clinostomum* spp. populations. Table 8: genetic to geographic distance comparison by Mantel test.
