# Supplementary material for: Genetic Diversity and Population Dynamics of Clinostomum spp. Using Comprehensive Bioinformatics Approaches
Source: Vet Med Int. 2024 Nov 23;2024:6924523. doi: 10.1155/vmi/6924523 (PMC11611402; doi:10.1155/vmi/6924523)
Supplement: Supporting Information — Additional supporting information can be found online in the Supporting Information section. [file 6924523.f1.docx]

**Table 1.** Sequence identity matrix (%) amongst the clinostomids based on the partial sequence of 18S rDNA gene.

| Scientific Name | Max Score | Total Score | Query Cover | Per. ident | | Accession |
| --- | --- | --- | --- | --- | --- | --- |
| *Clinostomum* sp*.* | 627 | 627 | 100% | 100 | | [**ON775556.1**](https://www.ncbi.nlm.nih.gov/nucleotide/ON775556.1?report=genbank&log$=nucltop&blast_rank=1&RID=CJ644R0X016) |
| *Clinostomum* sp*.* | 627 | 627 | 100% | 100 | | [**OP793994.1**](https://www.ncbi.nlm.nih.gov/nucleotide/OP793994.1?report=genbank&log$=nucltop&blast_rank=2&RID=CJ644R0X016) |
| *Clinostomum* sp. | 627 | 627 | 100% | 100 | | [**OP793993.1**](https://www.ncbi.nlm.nih.gov/nucleotide/OP793993.1?report=genbank&log$=nucltop&blast_rank=3&RID=CJ644R0X016) |
| *Clinostomum* sp*.* | 627 | 627 | 100% | 100 | | [**OP793992.1**](https://www.ncbi.nlm.nih.gov/nucleotide/OP793992.1?report=genbank&log$=nucltop&blast_rank=4&RID=CJ644R0X016) |
| *Clinostomum* sp*.* | 627 | 627 | 100% | 100 | | [**OP793991.1**](https://www.ncbi.nlm.nih.gov/nucleotide/OP793991.1?report=genbank&log$=nucltop&blast_rank=5&RID=CJ644R0X016) |
| *Clinostomum* sp*.* | 627 | 627 | 100% | 100 | | [**OP793990.1**](https://www.ncbi.nlm.nih.gov/nucleotide/OP793990.1?report=genbank&log$=nucltop&blast_rank=6&RID=CJ644R0X016) |
| *Clinostomum* sp*.* | 627 | 627 | 100% | 100 | | [**OP793989.1**](https://www.ncbi.nlm.nih.gov/nucleotide/OP793989.1?report=genbank&log$=nucltop&blast_rank=7&RID=CJ644R0X016) |
| *Clinostomum* sp*.* | 627 | 627 | 100% | 100 | | [**OP793988.1**](https://www.ncbi.nlm.nih.gov/nucleotide/OP793988.1?report=genbank&log$=nucltop&blast_rank=8&RID=CJ644R0X016) |
| *Clinostomum* sp. | 627 | 627 | 100% | 100 | | [**OP793987.1**](https://www.ncbi.nlm.nih.gov/nucleotide/OP793987.1?report=genbank&log$=nucltop&blast_rank=9&RID=CJ644R0X016) |
| *Clinostomum* sp. | 627 | 627 | 100% | 100 | | [**OP793986.1**](https://www.ncbi.nlm.nih.gov/nucleotide/OP793986.1?report=genbank&log$=nucltop&blast_rank=10&RID=CJ644R0X016) |
| *Clinostomum* sp. | 627 | 627 | 100% | 100 | | [**OP793985.1**](https://www.ncbi.nlm.nih.gov/nucleotide/OP793985.1?report=genbank&log$=nucltop&blast_rank=11&RID=CJ644R0X016) |
| *Clinostomum* sp. | 627 | 627 | 100% | 100 | | [**MW539004.1**](https://www.ncbi.nlm.nih.gov/nucleotide/MW539004.1?report=genbank&log$=nucltop&blast_rank=12&RID=CJ644R0X016) |
| *Clinostomid* sp*.* | 627 | 627 | 100% | 100 | | [**AY829252.1**](https://www.ncbi.nlm.nih.gov/nucleotide/AY829252.1?report=genbank&log$=nucltop&blast_rank=13&RID=CJ644R0X016) |
| *Clinostomum* sp. | 627 | 627 | 100% | 100 | | [**AY222094.1**](https://www.ncbi.nlm.nih.gov/nucleotide/AY222094.1?report=genbank&log$=nucltop&blast_rank=14&RID=CJ644R0X016) |
| *Clinostomum piscidium* | 608 | 608 | 100% | 99.12 | | [**FJ970655.1**](https://www.ncbi.nlm.nih.gov/nucleotide/FJ970655.1?report=genbank&log$=nucltop&blast_rank=15&RID=CJ644R0X016) |
| *Clinostomum brieni* | 586 | 586 | 100% | 97.94 | | [**MH606189.1**](https://www.ncbi.nlm.nih.gov/nucleotide/MH606189.1?report=genbank&log$=nucltop&blast_rank=16&RID=CJ644R0X016) |
| *Clinostomum brieni* | 586 | 586 | 100% | 97.94 | | [**MH606188.1**](https://www.ncbi.nlm.nih.gov/nucleotide/MH606188.1?report=genbank&log$=nucltop&blast_rank=17&RID=CJ644R0X016) |
| *Clinostomum complanatum* | 586 | 586 | 100% | 97.94 | | [**KF811012.1**](https://www.ncbi.nlm.nih.gov/nucleotide/KF811012.1?report=genbank&log$=nucltop&blast_rank=18&RID=CJ644R0X016) |
| *Clinostomum brieni* | 580 | 580 | 100% | 97.64 | | [**MH606187.1**](https://www.ncbi.nlm.nih.gov/nucleotide/MH606187.1?report=genbank&log$=nucltop&blast_rank=19&RID=CJ644R0X016) |
| *Clinostomum brieni* | 580 | 580 | 100% | 97.64 | | [**MH606186.1**](https://www.ncbi.nlm.nih.gov/nucleotide/MH606186.1?report=genbank&log$=nucltop&blast_rank=20&RID=CJ644R0X016) |
| *Clinostomum giganticum* | 579 | 579 | 98% | 97.91 | | [**FJ970654.1**](https://www.ncbi.nlm.nih.gov/nucleotide/FJ970654.1?report=genbank&log$=nucltop&blast_rank=21&RID=CJ644R0X016) |
| *Clinostomum complanatum* | 577 | 577 | 100% | 97.35 | | [**MK811210.1**](https://www.ncbi.nlm.nih.gov/nucleotide/MK811210.1?report=genbank&log$=nucltop&blast_rank=22&RID=CJ644R0X016) |
|  |  |  |  | |  |  |
| *Clinostomum complanatum* | 577 | 577 | 100% | 97.35 | | [**FJ609420.1**](https://www.ncbi.nlm.nih.gov/nucleotide/FJ609420.1?report=genbank&log$=nucltop&blast_rank=24&RID=CJ644R0X016) |
|  |  |  |  |  | |  |
| *Clinostomum brieni* | 575 | 575 | 100% | 97.35 | | [**KF811009.1**](https://www.ncbi.nlm.nih.gov/nucleotide/KF811009.1?report=genbank&log$=nucltop&blast_rank=26&RID=CJ644R0X016) |
| *Clinostomum cutaneum* | 569 | 569 | 100% | 97.05 | | [**GQ339114.1**](https://www.ncbi.nlm.nih.gov/nucleotide/GQ339114.1?report=genbank&log$=nucltop&blast_rank=27&RID=CJ644R0X016) |
| *Clinostomum cutaneum* | 569 | 569 | 100% | 97.05 | | [**FJ609421.1**](https://www.ncbi.nlm.nih.gov/nucleotide/FJ609421.1?report=genbank&log$=nucltop&blast_rank=28&RID=CJ644R0X016) |
| *Clinostomum poteae* | 558 | 558 | 100% | 96.47 | | [**MH282569.1**](https://www.ncbi.nlm.nih.gov/nucleotide/MH282569.1?report=genbank&log$=nucltop&blast_rank=29&RID=CJ644R0X016) |
| *Clinostomum poteae* | 558 | 558 | 100% | 96.47 | | [**MH282568.1**](https://www.ncbi.nlm.nih.gov/nucleotide/MH282568.1?report=genbank&log$=nucltop&blast_rank=30&RID=CJ644R0X016) |
| *Clinostomum poteae* | 558 | 558 | 100% | 96.47 | | [**MH282567.1**](https://www.ncbi.nlm.nih.gov/nucleotide/MH282567.1?report=genbank&log$=nucltop&blast_rank=31&RID=CJ644R0X016) |
| *Clinostomum phalacrocoracis* | 558 | 558 | 100% | 96.46 | | [**FJ609423.1**](https://www.ncbi.nlm.nih.gov/nucleotide/FJ609423.1?report=genbank&log$=nucltop&blast_rank=32&RID=CJ644R0X016) |
| *Clinostomum phalacrocoracis* | 558 | 558 | 100% | 96.46 | | [**FJ609422.1**](https://www.ncbi.nlm.nih.gov/nucleotide/FJ609422.1?report=genbank&log$=nucltop&blast_rank=33&RID=CJ644R0X016) |
| *Clinostomum marginatum* | 553 | 553 | 100% | 96.17 | | [**MF398350.1**](https://www.ncbi.nlm.nih.gov/nucleotide/MF398350.1?report=genbank&log$=nucltop&blast_rank=34&RID=CJ644R0X016) |
| *Clinostomum tataxumui* | 553 | 553 | 100% | 96.17 | | [**MF398349.1**](https://www.ncbi.nlm.nih.gov/nucleotide/MF398349.1?report=genbank&log$=nucltop&blast_rank=35&RID=CJ644R0X016) |
| *Clinostomum marginatum* | 553 | 553 | 100% | 96.17 | | [**KU708007.1**](https://www.ncbi.nlm.nih.gov/nucleotide/KU708007.1?report=genbank&log$=nucltop&blast_rank=36&RID=CJ644R0X016) |
| *Clinostomum marginatum* | 553 | 553 | 100% | 96.17 | | [**OQ271399.1**](https://www.ncbi.nlm.nih.gov/nucleotide/OQ271399.1?report=genbank&log$=nucltop&blast_rank=37&RID=CJ644R0X016) |
| *Clinostomum marginatum* | 553 | 553 | 100% | 96.17 | | [**HQ439560.1**](https://www.ncbi.nlm.nih.gov/nucleotide/HQ439560.1?report=genbank&log$=nucltop&blast_rank=38&RID=CJ644R0X016) |
| *Clinostomum marginatum* | 553 | 553 | 100% | 96.17 | | [**AY245760.1**](https://www.ncbi.nlm.nih.gov/nucleotide/AY245760.1?report=genbank&log$=nucltop&blast_rank=39&RID=CJ644R0X016) |
| *Clinostomum* sp. | 553 | 553 | 100% | 96.17 | | [**AY222095.1**](https://www.ncbi.nlm.nih.gov/nucleotide/AY222095.1?report=genbank&log$=nucltop&blast_rank=40&RID=CJ644R0X016) |
| *Clinostomum album* | 547 | 547 | 100% | 95.87 | | [**KU708008.1**](https://www.ncbi.nlm.nih.gov/nucleotide/KU708008.1?report=genbank&log$=nucltop&blast_rank=41&RID=CJ644R0X016) |

**Table 2.** Sequence identity matrix (%) amongst the clinostomids based on the partial sequence of ITS gene

| Species Name | Accession No. | E-value | Identity (%) |
| --- | --- | --- | --- |
| *Clinostomum piscidium* | KY312848 | 0.00 | 100 |
| *Clinostomum philippinense* | KP110570 | 0.00 | 99.52 |
| *Clinostomum sp.* | MT446431 | 0.00 | 99.52 |
| *Clinostomum sp.* | KY865653 | 0.00 | 98.25 |
| *Clinostomum tilapiae* | KY649356 | 0.00 | 98.25% |
| *Clinostomum phalacrocoracis* | KP110569 | 0.00 | 98.09% |
| *Clinostomum cutaneum* | KP110564 | 0.00 | 97.93% |
| *Euclinostomum heterostomum* | KY312847 | 0.00 | 97.46% |
| *Clinostomum chabaudi* | MW528863 | 0.00 | 96.98% |

**Table 3.** The sequences of *Clinostomum* spp. used in this study for genetic diversity analysis

| **Locations** | | Continents | | Accession Number | | Host | | |
| --- | --- | --- | --- | --- | --- | --- | --- | --- |
| Australia | | Oceania | | ON775556 | | Freshwater Snail | | |
| Australia | | Oceania | | MW539004 | | Murray Cod | | |
| USA | | North America | | AY829252 | | Freshwater Snails | | |
| USA | | North America | | AY222094 | | Firetail gudgeon | | |
| India | | Asia | | FJ970655 | | Banded gourami | | |
| Congo | | Africa | | MH606189 | | African Catfish | | |
| Congo | | Africa | | MH606188 | | African Catfish | | |
| India | | Asia | | KF811012 | | Asian Catfish | | |
| South Africa | | Africa | | MH606187 | | African Catfish | | |
| South Africa | | Africa | | MH606186 | | African Catfish | | |
| India | | Asia | | FJ970654 | | Snakehead | | |
| Italy | | Europe | | MK811210 | | European chub | | |
| China | | Asia | | MK490986 | | Red-crowned Crane | | |
| Italy | | Europe | | FJ609420 | | Grey herons | | |
| Israel | | Asia | | AY245701 | | Cormorant | | |
| India | | Asia | | KF811009 | | Asian Catfish | | |
| Kenya | | Africa | | GQ339114 | | Grey heron | | |
| Kenya | | Africa | | FJ609421 | | Nile tilapia | | |
| USA | | North America | | MH282569 | | Double-crested Cormorant | | |
| USA | | North America | | MH282568 | | Double-crested Cormorant | | |
| USA | | North America | | MH282567 | | Double-crested Cormorant | | |
| Kenya | | Africa | | FJ609423 | | Grey heron | | |
| Kenya | | Africa | | FJ609422 | | Nile tilapia | | |
| Mexico | | North America | | MF398350 | | Great egret | | |
| Mexico | | North America | | MF398349 | | Great egret | | |
| USA | | North America | | KU708007 | | Great egret | | |
| India | | Asia | | OQ271399 | | Snakehead | | |
| USA | | North America | | HQ439560 | | Salamander | | |
| USA | | North America | | AY245760 | | Freshwater Fish | | |
| Italy | | Europe | | AY222095 | | American bullfrog | | |
| USA | | North America | | KU708008 | | Great egret | | |
| Thailand | | Asia | | OP793985 | | Snakeskin gourami | | |
| Thailand | | Asia | | OP793986 | | Snakeskin gourami | | |
|  |  | |  | |  | |  |  |
|  |  | |  | |  | |  |  |

**Table 4** 18s rDNA genetic distances of the sequence used in the study

| Name | ID | ID | ID | ID | ID | ID | ID | ID | ID | ID | ID | ID | ID | ID | ID | ID | ID | ID | ID | ID | ID | ID | ID | ID | ID | ID | ID |
| --- | --- | --- | --- | --- | --- | --- | --- | --- | --- | --- | --- | --- | --- | --- | --- | --- | --- | --- | --- | --- | --- | --- | --- | --- | --- | --- | --- |
| OP793985 |  |  |  |  |  |  |  |  |  |  |  |  |  |  |  |  |  |  |  |  |  |  |  |  |  |  |  |
| OP793986 | 0.0 |  |  |  |  |  |  |  |  |  |  |  |  |  |  |  |  |  |  |  |  |  |  |  |  |  |  |
| OP793987 | 0.0 | 0.0 |  |  |  |  |  |  |  |  |  |  |  |  |  |  |  |  |  |  |  |  |  |  |  |  |  |
| OP793988 | 0.0 | 0.0 | 0.0 |  |  |  |  |  |  |  |  |  |  |  |  |  |  |  |  |  |  |  |  |  |  |  |  |
| OP793989 | 0.0 | 0.0 | 0.0 | 0.0 |  |  |  |  |  |  |  |  |  |  |  |  |  |  |  |  |  |  |  |  |  |  |  |
| OP793990 | 0.0 | 0.0 | 0.0 | 0.0 | 0.0 |  |  |  |  |  |  |  |  |  |  |  |  |  |  |  |  |  |  |  |  |  |  |
| OP793991 | 0.0 | 0.0 | 0.0 | 0.0 | 0.0 | 0. |  |  |  |  |  |  |  |  |  |  |  |  |  |  |  |  |  |  |  |  |  |
| OP793992 | 0.0 | 0.0 | 0.0 | 0.0 | 0.0 | 0.0 | 0.0 |  |  |  |  |  |  |  |  |  |  |  |  |  |  |  |  |  |  |  |  |
| OP793993 | 0.0 | 0.0 | 0.0 | 0.0 | 0.0 | 0.0 | 0.0 | 0.0 |  |  |  |  |  |  |  |  |  |  |  |  |  |  |  |  |  |  |  |
| OP793994 | 0.0 | 0.0 | 0.00 | 0.0 | 0.0 | 0.0 | 0.0 | 0.0 | 0.0 |  |  |  |  |  |  |  |  |  |  |  |  |  |  |  |  |  |  |
| KF811012 | 0.0173830362 | 0.0173830362 | 0.0173830362 | 0.0173830362 | 0.0173830362 | 0.0173830362 | 0.0173830362 | 0.0173830362 | 0.0173830362 | 0.0173830362 |  |  |  |  |  |  |  |  |  |  |  |  |  |  |  |  |  |
| MW539004 | 0.0000000000 | 0.0000000000 | 0.0000000000 | 0.0000000000 | 0.0000000000 | 0.0000000000 | 0.0000000000 | 0.0000000000 | 0.0000000000 | 0.0000000000 | 0.0173830362 |  |  |  |  |  |  |  |  |  |  |  |  |  |  |  |  |
| AY829252 | 0.0000000000 | 0.0000000000 | 0.0000000000 | 0.0000000000 | 0.0000000000 | 0.0000000000 | 0.0000000000 | 0.0000000000 | 0.0000000000 | 0.0000000000 | 0.0173830362 | 0.0000000000 |  |  |  |  |  |  |  |  |  |  |  |  |  |  |  |
| AY222094 | 0.0000000000 | 0.0000000000 | 0.0000000000 | 0.0000000000 | 0.0000000000 | 0.0000000000 | 0.0000000000 | 0.0000000000 | 0.0000000000 | 0.0000000000 | 0.0173830362 | 0.0000000000 | 0.0000000000 |  |  |  |  |  |  |  |  |  |  |  |  |  |  |
| FJ970655 | 0.0000000000 | 0.0000000000 | 0.0000000000 | 0.0000000000 | 0.0000000000 | 0.0000000000 | 0.0000000000 | 0.0000000000 | 0.0000000000 | 0.0000000000 | 0.0173830362 | 0.0000000000 | 0.0000000000 | 0.0000000000 |  |  |  |  |  |  |  |  |  |  |  |  |  |
| MH606189 | 0.0173830362 | 0.0173830362 | 0.0173830362 | 0.0173830362 | 0.0173830362 | 0.0173830362 | 0.0173830362 | 0.0173830362 | 0.0173830362 | 0.0173830362 | 0.0000000000 | 0.0173830362 | 0.0173830362 | 0.0173830362 | 0.0173830362 |  |  |  |  |  |  |  |  |  |  |  |  |
| MH606188 | 0.0173830362 | 0.0173830362 | 0.0173830362 | 0.0173830362 | 0.0173830362 | 0.0173830362 | 0.0173830362 | 0.0173830362 | 0.0173830362 | 0.0173830362 | 0.0000000000 | 0.0173830362 | 0.0173830362 | 0.0173830362 | 0.0173830362 | 0.0000000000 |  |  |  |  |  |  |  |  |  |  |  |
| KF811012 | 0.0173830362 | 0.0173830362 | 0.0173830362 | 0.0173830362 | 0.0173830362 | 0.0173830362 | 0.0173830362 | 0.0173830362 | 0.0173830362 | 0.0173830362 | 0.0000000000 | 0.0173830362 | 0.0173830362 | 0.0173830362 | 0.0173830362 | 0.0000000000 | 0.0000000000 |  |  |  |  |  |  |  |  |  |  |
| MK811210 | 0.0316219729 | 0.0316219729 | 0.0316219729 | 0.0316219729 | 0.0316219729 | 0.0316219729 | 0.0316219729 | 0.0316219729 | 0.0316219729 | 0.0316219729 | 0.0316697225 | 0.0316219729 | 0.0316219729 | 0.0316219729 | 0.0316219729 | 0.0316697225 | 0.0316697225 | 0.0316697225 |  |  |  |  |  |  |  |  |  |
| AY245701 | 0.0316219729 | 0.0316219729 | 0.0316219729 | 0.0316219729 | 0.0316219729 | 0.0316219729 | 0.0316219729 | 0.0316219729 | 0.0316219729 | 0.0316219729 | 0.0316697225 | 0.0316219729 | 0.0316219729 | 0.0316219729 | 0.0316219729 | 0.0316697225 | 0.0316697225 | 0.0316697225 | 0.0000000000 |  |  |  |  |  |  |  |  |
| KF811009 | 0.0209072771 | 0.0209072771 | 0.0209072771 | 0.0209072771 | 0.0209072771 | 0.0209072771 | 0.0209072771 | 0.0209072771 | 0.0209072771 | 0.0209072771 | 0.0034453169 | 0.0209072771 | 0.0209072771 | 0.0209072771 | 0.0209072771 | 0.0034453169 | 0.0034453169 | 0.0034453169 | 0.0317363476 | 0.0317363476 |  |  |  |  |  |  |  |
| FJ970654 | 0.0209072771 | 0.0209072771 | 0.0209072771 | 0.0209072771 | 0.0209072771 | 0.0209072771 | 0.0209072771 | 0.0209072771 | 0.0209072771 | 0.0209072771 | 0.0034453169 | 0.0209072771 | 0.0209072771 | 0.0209072771 | 0.0209072771 | 0.0034453169 | 0.0034453169 | 0.0034453169 | 0.0352451771 | 0.0352451771 | 0.0069085524 |  |  |  |  |  |  |
| MF398350 | 0.0351807518 | 0.0351807518 | 0.0351807518 | 0.0351807518 | 0.0351807518 | 0.0351807518 | 0.0351807518 | 0.0351807518 | 0.0351807518 | 0.0351807518 | 0.0316697225 | 0.0351807518 | 0.0351807518 | 0.0351807518 | 0.0351807518 | 0.0316697225 | 0.0316697225 | 0.0316697225 | 0.0388948284 | 0.0388948284 | 0.0281131433 | 0.0352451771 |  |  |  |  |  |
| MF398349 | 0.0280089815 | 0.0280089815 | 0.0280089815 | 0.0280089815 | 0.0280089815 | 0.0280089815 | 0.0280089815 | 0.0280089815 | 0.0280089815 | 0.0280089815 | 0.0245160055 | 0.0280089815 | 0.0280089815 | 0.0280089815 | 0.0280089815 | 0.0245160055 | 0.0245160055 | 0.0245160055 | 0.0463766651 | 0.0463766651 | 0.0209909768 | 0.0280595985 | 0.0209909768 |  |  |  |  |
| KX172121 | 0.0280089815 | 0.0280089815 | 0.0280089815 | 0.0280089815 | 0.0280089815 | 0.0280089815 | 0.0280089815 | 0.0280089815 | 0.0280089815 | 0.0280089815 | 0.0173984476 | 0.0280089815 | 0.0280089815 | 0.0280089815 | 0.0280089815 | 0.0173984476 | 0.0173984476 | 0.0173984476 | 0.0280595985 | 0.0280595985 | 0.0174322429 | 0.0209166198 | 0.0174322429 | 0.0174842989 |  |  |  |
| Z11979 | 0.0841049316 | 0.0841049316 | 0.0841049316 | 0.0841049316 | 0.0841049316 | 0.0841049316 | 0.0841049316 | 0.0841049316 | 0.0841049316 | 0.0841049316 | 0.0879099578 | 0.0841049316 | 0.0841049316 | 0.0841049316 | 0.0841049316 | 0.0879099578 | 0.0879099578 | 0.0879099578 | 0.0997685768 | 0.0997685768 | 0.0841049316 | 0.0917362756 | 0.0801679034 | 0.0761447091 | 0.0879099578 |  |  |
| MH606187 | 0.0209166198 | 0.0209166198 | 0.0209166198 | 0.0209166198 | 0.0209166198 | 0.0209166198 | 0.0209166198 | 0.0209166198 | 0.0209166198 | 0.0209166198 | 0.0034482895 | 0.0209166198 | 0.0209166198 | 0.0209166198 | 0.0209166198 | 0.0034482895 | 0.0034482895 | 0.0034482895 | 0.0353061021 | 0.0353061021 | 0.0069055383 | 0.0069055383 | 0.0280595985 | 0.0281131433 | 0.0209445618 | 0.0839573681 |  |
| MK490986 | 0.0316219729 | 0.0316219729 | 0.0316219729 | 0.0316219729 | 0.0316219729 | 0.0316219729 | 0.0316219729 | 0.0316219729 | 0.0316219729 | 0.0316219729 | 0.0389691222 | 0.0316219729 | 0.0316219729 | 0.0316219729 | 0.0316219729 | 0.0389691222 | 0.0389691222 | 0.0389691222 | 0.0069205257 | 0.0069205257 | 0.0390624860 | 0.0425713155 | 0.0388948284 | 0.0463766651 | 0.0353061021 | 0.0997685768 | 0.0426591758 |

**Table 5.** ITS genetic distances of the sequence used in the study

| Column1 |  |  |  |  |  |  |  |  |  |
| --- | --- | --- | --- | --- | --- | --- | --- | --- | --- |
| OP782661_Snakeskin_gourami_Thailand | ID |  |  |  |  |  |  |  |  |
| KY312848_Clinostomum_piscidium_India | 0.0063770876 | ID |  |  |  |  |  |  |  |
| KP110570_Clinostomum_philippinense_Thailand | 0.0095925327 | 0.0128287900 | ID |  |  |  |  |  |  |
| MT446431_Clinostomum_sp._Australia | 0.0095925327 | 0.0128287900 | 0.0000000000 | ID |  |  |  |  |  |
| KY865653_Clinostomum_sp._Italy | 0.0193253628 | 0.0226148483 | 0.0095925327 | 0.0095925327 | ID |  |  |  |  |
| KY649356_Clinostomum_tilapiae_Italy | 0.0209674004 | 0.0242677424 | 0.0112080430 | 0.0112080430 | 0.0047770862 | ID |  |  |  |
| KP110569_Clinostomum_phalacrocoracis_Canada | 0.0209878769 | 0.0242936741 | 0.0112203110 | 0.0112203110 | 0.0047789981 | 0.0063815635 | ID |  |  |
| KP110564_Clinostomum_cutaneum_Canada | 0.0226148483 | 0.0259261187 | 0.0128287900 | 0.0128287900 | 0.0063770876 | 0.0079822254 | 0.0079892820 | ID |  |
| MW528863_Clinostomum_chabaudi_Germany | 0.0343015239 | 0.0376913674 | 0.0243235491 | 0.0243235491 | 0.0177227932 | 0.0193648308 | 0.0161063433 | 0.0177227932 | ID |
| Z11979_Schistosoma_spindale_(Cattle_UK) | 0.5623392828 | 0.5559867769 | 0.5623392828 | 0.5623392828 | 0.5608412984 | 0.5608412984 | 0.5576618524 | 0.5687466014 | 0.5727466227 |

**Table 6.** The demographic expansion supported by the pairwise mismatch distribution analysis

| **Population Name** | **Mismatch observed mean** | **Mismatch observed variance** | **Tau** | **Theta0** | **Theta1** | **Test of goodness-of-fit** | |
| --- | --- | --- | --- | --- | --- | --- | --- |
|  |  |  |  |  |  | **Sum of Squared deviation** | **Harpending's Raggedness index** |
| Asia | 4.088 | 14.200 | 8.668 | 0.000 | 3.427 | 0.13635964 | 0.24762111 |
| Australia | 0.000 | 0.000 | - | - | - | - | - |
| Africa | 4.143 | 8.720 | 7.785 | 0.000 | 9.004 | 0.03422558 | 0.0612 |
| America | - | - | - | - | - | - | - |
| Europe | 6.667 | 33.333 | 11.133 | 0.004 | 99999.000 | 0.43663469 | 1.000 |

**Table 7.** Pairwise genetic differentiation among *Clinostomum* spp. populations

| Population | Population | Hs | Ks | Kxy | Gst | DeltaSt | GammaSt | Dxy | Da |
| --- | --- | --- | --- | --- | --- | --- | --- | --- | --- |
| Asia | Australia | 0.64052 | 40.85294 | 24.33333 | 0.09936 | 0.00156 | 0.01334 | 0.07285 | 0.0049 |
| Asia | America | 0.70448 | 41.60892 | 52.45454 | 0.0866 | 0.02103 | 0.15323 | 0.15705 | 0.03608 |
| Asia | Africa | 0.6996 | 32.87589 | 55.58333 | 0.13015 | 0.04093 | 0.30637 | 0.16642 | 0.09141 |
| Asia | Europe | 0.64206 | 43.85994 | 39.55556 | 0.13805 | 0.00481 | 0.0386 | 0.11843 | -0.00142 |
| Australia | America | 0.81818 | 29.96923 | 42 | 0.18344 | 0.02019 | 0.1984 | 0.12575 | 0.07273 |
| Australia | Africa | 0.85714 | 3.77143 | 49 | 0.24409 | 0.04497 | 0.81987 | 0.14671 | 0.13965 |
| Australia | Europe | 0.66667 | 20.8 | 22.66667 | 0.447 | 0.01597 | 0.27778 | 0.06786 | 0.01597 |
| America | Africa | 0.83377 | 22.49022 | 32.52273 | 0.08774 | 0.02096 | 0.25572 | 0.09737 | 0.03729 |
| America | Europe | 0.80303 | 35.25714 | 39.90909 | 0.08515 | 0.01235 | 0.12003 | 0.11949 | 0.01457 |
| Africa | Europe | 0.82993 | 12.88312 | 51.16667 | 0.12356 | 0.0446 | 0.61555 | 0.15319 | 0.09424 |

Hs (average genetic diversity within populations)

Ks (number of synonymous substitutions per synonymous site)

Kxy (number of nonsynonymous substitutions per nonsynonymous site)

Gst (genetic differentiation among populations)

DeltaSt (differentiation between subpopulations within populations)

GammaSt (differentiation between populations within the continents)

Dxy (genetic distance between populations)

Da (difference between Kxy and Ks)

**Table 8.** Genetic to geographic distances comparison by Mantel test

| Features | Value/Remark |
| --- | --- |
| Mantel statistic (r) | -0.003764 |
| Significance (P>0.05) | 0.49451 (Not Significant) |
